# Emotional scars: limbic brain processing alterations in adults with childhood abuse across mental health disorders

**DOI:** 10.1038/s41380-026-03511-9

**Published:** 2026-03-05

**Authors:** Mayuresh S. Korgaonkar, Cheryl Tobler, Kim Felmingham, Leanne M. Williams, Richard A. Bryant, Isabella A. Breukelaar

**Affiliations:** 1 Brain Dynamics Centre, Westmead Institute for Medical Research, University of Sydney, Westmead, NSW Australia; 2 Discipline of Psychiatry, Sydney Medical School, Westmead, NSW Australia; 3 School of Psychology, University of New South Wales, Sydney, Australia; 4 School of Psychological Sciences, University of Melbourne, Parkville, Australia; 5 Department of Psychiatry and Behavioral Sciences, Stanford University, Palo Alto, USA; 6 Sierra-Pacific Mental Illness Research, Education, and Clinical Center (MIRECC) VA Palo Alto Health Care System, Palo Alto, USA

**Keywords:** Psychiatric disorders, Psychology

## Abstract

Abuse experienced during childhood and adolescence significantly influences brain development and increases the risk of psychiatric disorders later in life. However, its long-term impact on emotion processing across psychiatric conditions remains underexplored. In this functional MRI study, we examined a sample of 635 individuals with and without childhood abuse, including people with depression, anxiety disorders, PTSD, and healthy controls, to investigate differences in brain activation during conscious and non-conscious emotional face processing. Brain activation across regions involved in emotional regulation was compared in response to negative (anger, fear, disgust, sadness), happy and neutral facial expressions. Individuals exposed to abuse before age 13 showed heightened hippocampal activation during non-conscious negative emotion processing compared to non-abused individuals, an effect absent in those exposed to abuse between ages 13 and 18. Additionally, amygdala activation during conscious emotion processing was elevated across all emotions in individuals who experienced adolescent abuse (13–18 years), compared to both abuse-free individuals and those exposed to early childhood abuse. This transdiagnostic approach highlights distinct vulnerability windows in brain development, with differential effects on emotion processing depending on the timing of abuse. Our study provides novel insights into how early life adversities shapes emotional processing, advancing our understanding of its transdiagnostic impact on brain function.

## Introduction

Childhood and adolescence are crucial stages of development where experiences can have a lasting impact on psychological well-being. Adverse experiences such as abuse during these formative years can significantly affect how individuals process and regulate emotions [1]. These effects are profound, influencing both conscious and non-conscious emotional processes and contributing to the development of various adult psychiatric disorders, including depression, anxiety, and stress-related conditions [2–4].

Research employing functional magnetic resonance imaging (fMRI) has significantly advanced our understanding of the neural mechanisms underpinning these impacts. Studies have shown that childhood adversity can cause structural and functional alterations in brain regions implicated in emotion processing, such as the amygdala, prefrontal cortex, and hippocampus in otherwise healthy individuals [5, 6]. Consistent findings indicate that individuals with a history of childhood abuse exhibit heightened amygdala reactivity to emotional stimuli [7]. This is often accompanied by decreased dorsolateral prefrontal cortex activity [8] and increased activation in the anterior insula, rostral medial prefrontal cortex and dorsal anterior cingulate cortex (ACC) during emotion processing [9]. These neural alterations collectively appear to underlie emotional regulation difficulties observed in this population [8, 10].

Despite significant advances in understanding the neural functions associated with childhood abuse, there is still much to explore regarding its long-term effects on brain systems. A strong foundation in the literature has established a link between early life abuse and altered limbic activity, increasing vulnerability to major psychiatric disorders such as major depressive disorder (MDD), posttraumatic stress disorder (PTSD), and anxiety disorders [10–12]. While numerous studies have examined these long-term effects, particularly in adolescence[13] and early adulthood [14, 15], emerging research offers the opportunity to address variability in findings by systematically investigating the timing of abuse and its effects on distinct neural pathways. For example, research differentiates between the impacts of abuse on conscious versus non-conscious emotion processing [16]. Conscious emotion processing, which involves the deliberate appraisal and regulation of emotional stimuli, engages an *indirect* amygdala pathway that involves cortical processing [17]. In contrast, non-conscious processing engages the *direct* amygdala pathway bypassing cortical sensory processing and activating automatic, fight-or-flight responses without conscious or verbal input [17]. Understanding how the timing of abuse affects these pathways has significant implications for treatment and recovery [18–20]. While there is increasing support for the idea that there are sensitive periods in neural development when abuse may be most detrimental [4, 21], a recent systematic review by Schaefer and colleagues [22] highlighted that empirical evidence remains limited and inconsistent, largely due to methodological variability across studies, coarse age categorizations, inconsistent definitions of maltreatment, and variability in clinical endpoints. As a result, the field lacks clear consensus on when during development abuse is most neurobiologically disruptive. To address this, our study examines whether abuse occurring before versus after age 13—a transitional period marking major neurocognitive and emotional development—differentially affects neural circuits involved in conscious and non-conscious emotion processing. Specifically, abuse occurring in early childhood (ages 0–5) may be harder to overcome than abuse in later adolescence[19], as older individuals have more developed cognitive capacities for conscious emotional processing, allowing them to contextualize and verbally process traumatic experiences, while younger children may be less able to do so leaving emotional scars unprocessed at a conscious level [17, 18, 23, 24].

Additionally, studying these effects in a transdiagnostic sample that includes various disorders linked to early life abuse could provide novel insights into whether a common neural mechanism, involved in emotion regulation, is activated or exacerbated by the chronic stress of childhood abuse. This would help disentangle the direct effects of abuse from those of associated psychopathological conditions, shedding light in the underlying mechanisms that contribute to heightened vulnerability across different psychiatric disorders [25].

To advance our understanding of the long-term neural effects of childhood abuse, this study investigates how abuse experienced in childhood and adolescence affects both conscious and non-conscious emotion processing in a transdiagnostic sample of individuals with depression, anxiety, and stress disorders. By employing fMRI paradigms that separately assess conscious and non-conscious processing, we aim to delineate the common and distinct neural signatures associated with childhood maltreatment across these two pathways. Prior work has indicated that the timing of abuse plays a critical role in determining its impact on neural development, with sensitive periods occurring predominantly by the age of 14 [4, 26]. Accordingly, we examined whether abuse-related functional alterations in the brain differed based on whether the abuse occurred before or after the age of 13, exploring how this timing influences direct versus indirect pathways involved in emotional regulation.

## Materials and methods

### Participants

All procedures were approved by Western Sydney Area Health Service Human Ethics Committee. Written informed consent was obtained from all participants. All methods were performed in accordance with the relevant guidelines and regulations. There were initially 808 adult participants (aged 18–65 years) recruited and tested from 2009–2015 at the Brain Dynamics Centre at Westmead Institute for Medical Research, Sydney, Australia. Participants were recruited as part of three overlapping studies through community advertisements and referral from clinicians to participants in research on neural markers of depression, anxiety, and as healthy controls. Details of these studies are provided in the supplementary materials (Table S1).

Mental health status was determined by a structured clinical interview (based on DSM-IV) using the Mini International Neuropsychiatric Interview (MINI version 5.5) [27]. In our cohort (across the three studies), participants were diagnosed into major depressive disorder (MDD), general anxiety disorder, panic disorder, social phobia, complicated grief, complex and standard posttraumatic stress disorder (PTSD), mild traumatic brain injury, trauma experienced healthy individuals, healthy individuals who experienced grief (grief controls) and healthy individuals with no exposure to trauma or grief. For our analysis, we categorised participants broadly into depression (MDD, complicated grief), anxiety (general anxiety disorder, panic disorder, social phobia), stress (standard and complex PTSD) and controls (trauma, grief and healthy individuals with no trauma or grief exposure).

Participants were recruited through advertisements to participate in clinical research or treatment trials. Exclusion criteria for participation was a history of neurological disorder, psychosis, or current substance dependence. Participants on a psychotropic medication were eligible to be included if they were on a stable dosage for at least two months prior to testing.

Participants were assessed on symptom measures including the DASS (Depression and Anxiety Stress Scale [28] to assess levels of depression, anxiety, and stress and Early Life Stress Questionnaire (ELSQ) [29] to assess adverse childhood and adolescent events. Within the ELSQ, participants were asked if they had experienced sexual abuse, physical abuse, or emotional abuse (each separately) and indicate the age bracket at which the abuse first occurred i.e. either within 0–3 years, 4–7 years, 8–12 years, or 13–17years.

Data for 119 individuals was excluded either due to not meeting study criteria (n = 5), artefacts present on MRI scans (n = 3), not completing fMRI tasks (n = 34), too much movement on fMRI scans (n = 77). Further, 15 participants were under 18 at the time of completing the study, 39 participants did not complete the ELSQ questionnaire, resulting in data from total 635 individuals.

Our primary analysis compared all individuals who experienced abuse before the age of 18 versus those who did not. Due to strong prior evidence of neural and psychological correlates of child abuse being distinctly manifested pre-adolescence [19], we also performed a secondary analysis on only those who had experienced abuse prior to 13 years of age or post 13 years (13–18years of age) respectively, comparing them to each other and to non-abused participants separately.

### Emotional faces processing task

All participants completed a conscious and nonconscious emotion processing fMRI task which involved the passive viewing of 6 emotions (fear, angry, sad, disgust, happy and neutral, displayed on eight different faces [30] (4 males, 4 females)) presented pseudorandomly for 500 ms (conscious) and 10 ms (non-conscious) in blocks of 8, repeated 5 times for a duration of 5 min per task. The duration of face presentation was chosen based on psychophysiological evidence that emotional faces can be differentiated at ≤ 20 ms and explicitly identified at ≥ 330 ms [31, 32]. There was a 1233.3 ms interstimulus interval for the nonconscious task, and a 750 ms interstimulus interval for the conscious task. For the nonconscious task, each emotional face was superseded by the presentation of a 150 ms neutral face that was randomly oriented one degree from the two-dimensional plane to reduce conscious discrimination based on facial features. The nonconscious task was always presented first to reduce priming effects induced by conscious viewing of faces (conscious condition) on processing of nonconscious faces. To motivate participants to maintain concentration on the faces we instructed them that they would be asked post-scan questions about the faces. Full task parameters are described in Korgaonkar et al 2013 and in Supplementary Table s2 and Figure s1 [31].

### fMRI acquisition & pre-processing

Functional MRI data was acquired on a single 3 T GE Signa Twinspeed HDxT MR Scanner (GE Healthcare, Milwaukee, WI) using an 8-channel phased-array head coil (full acquisition details are in the supplementary methods). Standard pre-processing steps were performed using Statistical Parametric Mapping (SPM12; Wellcome Department of Cognitive Neurology) and FSL5 (FMRIB Software Library, University of Oxford) and involved image realignment and warping, normalization, and signal estimation via extraction of white matter and cerebral spinal fluid and smoothing using a 8 mm Gaussian Kernel. Scrubbing was performed on volumes, as well as the volume immediate proceeding it, that exceeded a fractional displacement of 0.3 mm or a difference in signal intensity greater than 10 from one volume to the next. Participant were excluded for excessive motion if the total number of scrubbed volumes exceeded more than 1/3 of the total scan time. Mean framewise displacement was calculated for each participant and participants were excluded if the average was greater than 0.2 mm across the whole scan. The pre-processing protocol has been described in more detail in previous work [33–35]. During first level analyses, neural data from each emotion block were transformed into a Blood Oxygenation Level-Dependent (BOLD) response using a standard canonical hemodynamic response function within a general linear model framework. Data was high pass filtered at 1/128 Hz cutoff to remove slow drifts and low-frequency noise from the BOLD signal. Motion parameters were also included as covariates in the first level model. Contrast images were generated for each emotion versus implicit rest baseline. These images were used to investigate neural differences associated with emotional processing between groups at the second-level of analysis.

### fMRI analysis

For each version of the task (conscious and non-conscious), we performed an ANOVA to compare participants who experienced abuse before the age of 18 to participants who had no experienced abuse using a voxelwise 2 × 6 flexible factorial repeated measures design in SPM 12. Group was the between-subjects factor and emotion type the within subject-factor. Using this model, F-contrasts were produced to investigate the main effect of group and interaction between group and emotion. As there were groups differences in age, sex, years of education and clinical diagnosis (Table 1), these measures were mean-centred and included as covariates of no interest. Motion was not included as a covariate of no interest at this level due to correction during first level processing but was checked post-hoc in any relevant analyses. A region of interest (ROI) analysis approach was used. The following ROIs were selected based on their involvement in emotional processing from existing literature and as used in our previous work [36]: the bilateral amygdala, bilateral hippocampus and bilateral insula, were generated using the Automated Anatomical Labelling (AAL) atlas [37], whereas the dorsal, pregenual and subgenual anterior cingulate cortex, dACC: 0 24 38; pgACC: 0 42 4, sgACC: 0 24 –8) [38], and the bilateral dorsolateral prefrontal cortex (DLPFC, L: −36 20 26, R: 46 30 18) [39] were constructed using a 8 mm radius sphere from *meta*-analyses of fMRI studies on emotional processing for greater and consistent activation for emotion processing versus baseline (either fixation cross or neutral). Voxelwise effects were evaluated to be significant at a family wise error corrected p < 0.05.

**Table 1:** Demographic and clinical characteristics by abuse status and age at exposure.

	Not Abused	Abused	P-value for no abuse vs abuse	Abused under 13	P-value for no abuse vs abuse at < 13 yrs of age	Abused over 13	P-value for no abuse vs abuse at > 13 yrs of age	P-value for abuse at < 13 yrs vs > 13 yrs	Total
	(N = 399)	(N = 236)		(N = 162)		(N = 72)			(N = 635)
**Age, y**									
Mean (SD), [Min, Max]	31.8 (11.1), [18.2, 69.2]	35.4 (12.8), [18.3, 65.2]	<0.001^^^	37.3 (12.9), [18.3, 65.2]	<0.001^^^	30.9 (11.5), [18.5, 62.3]	0.518^^^	<0.001^^^	33.1 (11.9), [18.2, 69.2]
**Sex**									
FEMALE	202 (50.6%)	145 (61.4%)	0.0104^*^	99 (61.1%)	0.031^*^	44 (61.1%)	0.131^*^	1*	347 (54.6%)
MALE	197 (49.4%)	91 (38.6%)		63 (38.9%)		28 (38.9%)			288 (45.4%)
**Years of education**									
Median [Min, Max]	16.0 [1.00, 18.0]	14.0 [3.00, 18.0]	0.003^^^	14.0 [3.00, 18.0]	0.002^^^	15.0 [10.0, 18.0]	0.380^^^	0.119^^^	15.0 [1.00, 18.0]
**Primary diagnosis (num, %)**									
General anxiety	6 (1.5%)	4 (1.7%)	<0.001^*^	3 (1.9%)	<0.001^*^	1 (1.4%)	<0.001^*^	0.006^*^	10 (1.6%)
Complicated grief	8 (2.0%)	12 (5.1%)		9 (5.6%)		3 (4.2%)			20 (3.1%)
Healthy control	180 (45.1%)	35 (14.8%)		26 (16.0%)		9 (12.5%)			215 (33.9%)
complex PTSD	1 (0.3%)	26 (11.0%)		25 (15.4%)		0 (0%)			27 (4.3%)
Grief control	17 (4.3%)	4 (1.7%)		3 (1.9%)		1 (1.4%)			21 (3.3%)
MDD	113 (28.3%)	102 (43.2%)		57 (35.2%)		44 (61.1%)			215 (33.9%)
mTBI	19 (4.8%)	6 (2.5%)		5 (3.1%)		1 (1.4%)			25 (3.9%)
Panic disorder	9 (2.3%)	12 (5.1%)		8 (4.9%)		4 (5.6%)			21 (3.3%)
PTSD	17 (4.3%)	18 (7.6%)		16 (9.9%)		2 (2.8%)			35 (5.5%)
Social Phobia	14 (3.5%)	11 (4.7%)		7 (4.3%)		4 (5.6%)			25 (3.9%)
Trauma control	15 (3.8%)	6 (2.5%)		3 (1.9%)		3 (4.2%)			21 (3.3%)
**Abuse type (num, %)**									
**Physical**			NA		NA		NA		
No	399 (100%)	110 (46.6%)		64 (39.5%)		46 (63.9%)		<0.001^*^	509 (80.2%)
Yes	0 (0%)	126 (53.4%)		98 (60.5%)		26 (36.1%)			126 (19.8%)
**Sexual**			NA		NA		NA		
No	399 (100%)	146 (61.9%)		88 (54.3%)		57 (79.2%)		<0.001^*^	545 (85.8%)
Yes	0 (0%)	89 (37.7%)		74 (45.7%)		15 (20.8%)			89 (14.0%)
**Emotional**			NA		NA		NA		
No	399 (100%)	43 (18.2%)		28 (17.3%)		15 (20.8%)		0.643*	442 (69.6%)
Yes	0 (0%)	192 (81.4%)		134 (82.7%)		57 (79.2%)			192 (30.2%)
**Extreme poverty or neglect**			NA		NA		NA		
No	399 (100%)	169 (71.6%)		107 (66.0%)		61 (84.7%)		0.006^*^	568 (89.4%)
Yes	0 (0%)	66 (28.0%)		55 (34.0%)		11 (15.3%)			66 (10.4%)
**DASS score, mean (SD),**									
Depression	10.4 (11.2)	19.2 (12.1)	<0.001^^^	18.7 (12.4)	<0.001^^^	20.2 (11.6)	<0.001^^^	0.377^^^	13.7 (12.3)
Anxiety	5.79 (7.00)	11.2 (8.40)	<0.001^^^	11.8 (9.04)	<0.001^^^	9.59 (6.65)	<0.001^^^	0.036^^^	7.79 (7.98)
Stress	11.2 (9.46)	18.6 (10.3)	<0.001^^^	18.9 (10.7)	<0.001^^^	18.1 (9.50)	<0.001^^^	0.570^^^	14.0 (10.4)
**Scan Motion Parameters**									
**Non-conscious face viewing**									
FD (movement), mean (SD)	0.0756 (0.0379)	0.0821 (0.0371)	0.0346^^^	0.0834 (0.0368)	0.0256^^^	0.0802 (0.0380)	0.344^^^	0.558^^^	0.0780 (0.0377)
**Conscious face viewing**									
FD (movement), mean (SD)	0.0792 (0.0382)	0.0833 (0.0381)	0.190	0.0865 (0.0388)	0.0454^^^	0.0772 (0.0358)	0.664^^^	0.078^^^	0.0808 (0.0382)

Participant characteristics for the total sample (N = 635) stratified by no abuse (N = 399) and any abuse (N = 236), with the abused group further divided by age at first exposure ( < 13 years, N = 162; ≥13 years, N = 72). Continuous variables are presented as mean (SD) or median [range]; categorical variables as n (%). P-values reflect comparisons between groups as indicated. Two-tailed t-tests were used for continuous variables and χ² tests for categorical variables.

*DASS* depression and anxiety stress scale, *FD* mean framewise displacement for entire scan, *MDD* major depression disorder, *mTBI* mild trauamtic brain injury, *NA* not applicable, *PTSD* posttraumatic stress disorder.

^ Two-tailed *t* test.

* X2 Test.

Given prior evidence that neural changes following abuse may be specific to abuse occurring in childhood vs adolescence, a secondary analysis was repeated in SPM12 using the same flexible factorial design as in the primary analysis but comparing a) participants who experienced abuse before 13 years of age with non-abused participants, b) participants who experienced abuse after 13 years of age with non-abused participants, and c) participants who experienced abuse before and after adolescence, controlling for the same covariates as above.

Post-hoc ANCOVAs were performed using identified significant clusters for main effects of group and group*emotion interaction effects to interpret the neural differences for each emotion type between groups controlling for same variables as in the SPM analysis (age, sex, years of education and clinical diagnosis). To do this, we extracted mean beta estimates for significant clusters and analysed differences between the group for all possible differences between emotions across the two groups. This was done by subtracting mean cluster activation between each of the emotions to create contrast variables between each of the emotions (i.e. Anger – Disgust, Anger – Fear, Anger – Happy).

Further post-hoc tests were performed to examine if the observed effects were consistent across diagnostic groups (depression, anxiety and stress). An ANCOVA analysis was performed using the significant clusters from the previous analysis as dependent variables, and abuse group, diagnosis group and abuse group*diagnosis interaction as independent variables (controlling for age, sex, years of education). For any significant interaction effects, we conducted posthoc comparisons between abuse vs non-abuse groups within each diagnostic group.

We also tested for group differences and interactions of emotional responses with sex for the identified significant clusters using an ANCOVA (controlling for age, years of education, clinical group) as well as associations with abuse type, load and abuse onset. We also assessed if the identified effects were driven by current symptom scores by testing the association between mean activation of significant clusters and DASS scores and also by testing if the abuse related effects survive controlling for DASS scores.

## Results

### Participant demographics

Six-hundred and thirty-five participants (mean age, 33.1 ± 11.9, 55% female, 235 diagnosed with MDD, 56 with anxiety disorders, 62 with PTSD and 282 healthy individuals) were included in the analysis for this study after exclusion and quality checking. This included 399 participants with no experience of abuse or neglect (mean age, 31.8 ± 11.1, 51% female) and 236 participants who reported a history of emotional, physical, or sexual abuse or neglect (mean age, 35.4 ± 12.8, 61% female) prior to 18 years of age (Table 1). The non-abused and abused groups differed on age (abuse>non-abuse; mean diff, −3.57, t = −3.58, p < 0.001) and years of education (abuse<non-abuse; mean diff, +0.69, *t* = 2.97, *p* = 0.003). The abused group also had higher motion during scanning (mean framewise displacement; mean diff, −0.007, *t* = 2.12, *p* = 0.035) on the non-conscious face processing task. There were 10% more females in the abused group (χ^2^ = 6.57, *p* = 0.01) as well as greater proportion of individuals with psychiatric diagnosis (80%) compared to the non-abused group (42%; χ^2^ = 77.62, *p* <0.001); specifically, a higher proportion of individuals diagnosed with PTSD and MDD in the abused group. The group that had experienced abuse also had higher levels of anxiety ( + 5.37 DASS score, *t* = 8.25, *p* < 0.001), depression ( + 8.76 DASS score, t = 9.04, p < 0.001) and stress ( + 7.43 DASS score, *t* = 9.02, *p* < 0.001) symptoms at time of testing. There was a high degree of comorbidity across diagnostic groups with 83.9% of individuals with a primary diagnosis of PTSD also meeting MINI criteria for other anxiety disorders and 71% for MDD, 54% of individuals with a primary diagnosis of MDD also presented with non-PTSD anxiety disorders and 55.4% of individuals with a non-PTSD anxiety disorder also meeting criteria for MDD (table s3 and figure s2).

Of the abused participants, 162 (68.6%) had experienced abuse prior to 13 years of age whereas 72 had experienced abuse after the age of 13 (Table 1). The abuse under 13 group was older (mean diff, +6.6 yrs, *t* = 3.80, *p* < 0.001) and had greater DASS anxiety (mean diff, +2.2, *t* = 2.11, *p* = 0.036) than the over 13 abuse group. While there was no difference in total number of individuals with a diagnosis (χ^2^ = 0.17, *p* = 0.68), they did differ on proportion of diagnostic groups (χ^2^ = 24.67, *p* = 0.006), specifically the under 13 abuse group had higher proportion of both complex (15 vs 0%) and standard PTSD (10 vs 3%) and lower proportion of MDD (35 vs 61%) compared to those who experienced abuse after 13. The under 13 abuse group had experienced more physical (χ^2^ = 10.92, *p* < 0.001) and sexual abuse (χ^2^ = 12.02, *p* < 0.001) and more neglect (χ^2^ = 7.69, *p* = 0.006) compared to the over 13 abuse sufferers.

### Abuse-related alterations in neural response to emotion processing

#### Non-conscious emotional processing

**Fig. 1:**
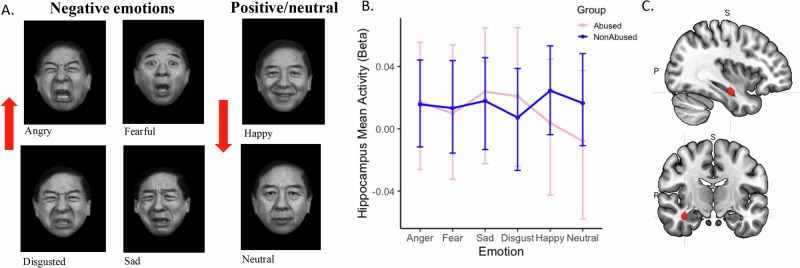
Hippocampal reactivity to non-conscious processing of emotional faces in adults who experienced childhood abuse. There was a significant group by emotion interaction in the right hippocampus when comparing those abused before age 18 with non-abused participants (MNI coordinates X = 38, Y = −12, Z = −20, K (cluster size) =57, Peak z-score = 4.48, *p* = 0.003 (FWE-corr). Post-hoc tests of extracted beta-values for hippocampal region found to be significant (**C**) identified this was due to a combination of increased hippocampal reactivity in response to negative emotions and decreased hippocampal response to positive and neutral emotions in abused participants (**A** & **B**). Error bars represent 95% confidence intervals.

When comparing all adults who experienced abuse to non-abused adults, there were no significant main effects of group (abuse vs non-abuse). However, there was a significant group by emotion interaction in the right hippocampus (MNI coordinates X = 38, Y = −12, Z = −20, K (cluster size) =57, peak z-score=4.48, *p*_FWE_ = 0.003 [Fig. 1]. Post-hoc tests indicated this was driven by increased hippocampal activity in those that experience abuse for negative emotions (angry, sad, disgust, fear) compared to happy and neutral faces (Table s2), specifically for contrasts comparing Anger v Happy [*p* = 0.024], Disgust v Happy [*p* = 0.001], Sad v Happy [*p* = 0.014]) and Anger v Neutral [*p* = 0.012], Disgust v Neutral [*p* < 0.001], Fear v Neutral [*p* = 0.040], Sad v Neutral [*p* = 0.004] emotions [Fig. 1, Table s4].

**Fig. 2:**
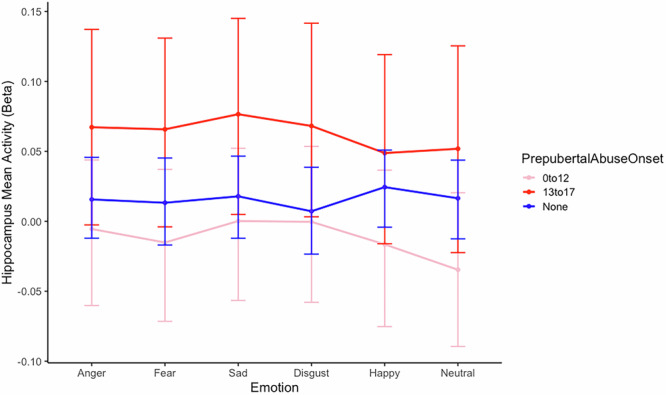
Hippocampal activity during non-conscious processing of different emotional faces for individuals who experienced abuse before and after the age of 13 and those with no abuse experience. Only those who experienced abuse prior to 13years of age had a differential activation profile compared to individuals with no experience of abuse. Error bars represent 95% confidence intervals.

When the abused group was divided into those who experienced abuse before and after the age of 13 compared to non-abused participants, the hippocampal result above remained significant for those that experienced abuse prior to 13years (MNI coordinates, X = 36, y = −10, Z = −22, k = 79, peak-z = 4.59, *p*_FWE_ = 0.002) but not for those who experienced abuse post 13years (Fig. 2). However, there were no activation differences when directly comparing the pre vs. post 13 abuse groups.

#### Conscious emotional processing

There was no difference between the two primary groups i.e. all adults who experienced abuse compared to non-abused adults (main effect of group or group*emotion interactions) for the conscious face viewing task.

**Fig. 3:**
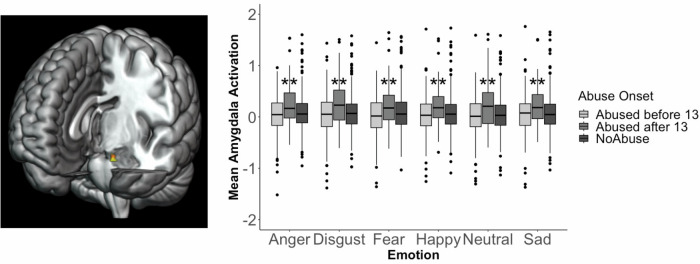
Amygdala activity in response to conscious processing of emotional faces in adults who experienced childhood abuse. Amygdala was significantly more active in participants who experienced abuse between the aged of 13 and 18 than those that experienced abuse preadolescence (YELLOW region; MNI coordinates, X = −12, Y = −8, Z = −22, k = 17, peak-Z = 4.48, *p* = 0.002(FWE-corr)) or no abuse (ORANGE region; MNI coordinates, X = −12, Y = −8, Z = −20 k = 6, peak-Z = 4.2, *p* = 0.009 (FWE-corr)). There were no significant differences between those who experienced abuse under 13 years and those who did not experience childhood abuse.

However, participants who experienced abuse after the age of 13 displayed heightened amygdala reactivity across all emotions (main effect of group; MNI coordinates, X = −12, Y = −8, Z = −20 k = 6, peak-z = 4.2, *p*_FWE_ = 0.009) but there were no significant differences in those abused before the age of 13 as compared with the non-abused group. This effect remained significant when comparing the two abuse groups directly with greater amygdala activation for the post-13 abused group (main effect of group; MNI coordinates, X = −12, Y = −8, Z = −22, k = 17, peak-z = 4.48, *p*_FWE_ = 0.002). Poc-hoc tests using extracted beta-values for this region confirmed significantly higher amygdala reactivity across all emotions (Fig. 3 and Table s7).

### Post-hoc analyses

Next, we investigated in series of *posthoc* analyses if these identified neural effects are common or different across diagnostic groups (depression, anxiety and stress); across sex; dependent on abuse type and load; and if driven by current clinical symptoms.

#### Effects of diagnosis and current symptoms

In an ANCOVA analysis, we tested for significant diagnosis*abuse group interaction effects on both hippocampal activation during non-conscious task and amygdala activation during the conscious task (controlling for age, sex, years of education). Current diagnosis did not have a significant effect on the relationship between abuse and hippocampal activation for non-conscious emotion processing. There were also no activation differences between the diagnostic categories overall for any of the emotion contrasts (i.e. main effect of diagnosis). This remained true even when analyses were performed using groups of differing abuse onset ages.

In contrast, there was a significant diagnosis*abuse group interaction effect for amygdala activation during conscious emotion processing for all emotions when comparing both abuse before the age of 13 to abuse after 13 as well as comparing abuse after 13 to no abuse (Table s8). Posthoc analyses found that this was due to higher amygdala activation across all emotions in adolescent-onset abuse with PTSD compared to healthy and anxious with abuse onset at the same time (p < 0.05, Table s9). Additionally adolescent-onset abuse vs non-abuse and childhood-onset abuse were found to have greater amygdala activation across all emotions only in those that also had a diagnosis of MDD and stress groups (p < 0.05, Table s10, Figure s3).

To evaluate if current clinical symptoms (as measured with DASS) were driving hippocampal and amygdala activation differences between abuse groups, we tested if these differences survived controlling for DASS measures in an ANCOVA analysis (in addition to controlling age, sex, years of education and diagnostic group). Both neural differences persisted despite controlling for symptom measures. We also tested if any of the symptom measures (depression, anxiety or stress) were correlated with neural activation in the abuse cohort and found no significant associations further confirming that the neural effects observed were unrelated to current symptom measures.

#### Sex effects

**Fig. 4:**
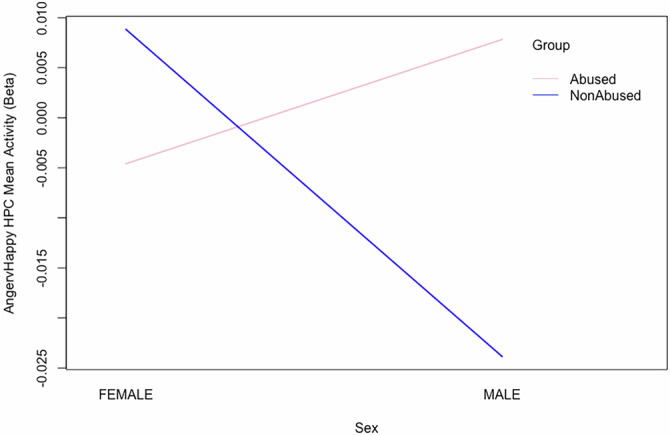
Differences in hippocampal reactivity to non-conscious facial emotions in abused and non-abused males and females. There was a significant interaction for the angry vs happy mean hippocampal (HPC) activity with sex (F = 9.29, *p* = 0.002) which was driven by higher hippocampal reactivity to this contrast in abused males compared to non-abused males but lower hippocampal reactivity to this contrast in abused females compared to non-abused females.

In an ANCOVA analysis, we tested for significant main effect of sex or sex*abuse group interaction on both neural activations controlling for age, years of education and diagnostic group. There were no significant differences between males vs. females (main effect of sex) for either the hippocampus activation for non-conscious emotions or the amygdala for conscious emotion processing. However, there was a significant abuse group*sex interaction for the angry vs happy contrast for the non-conscious task (*F* = 9.29, *p* = 0.002) which was driven by higher hippocampal reactivity to this contrast in abused males compared to non-abused males but lower hippocampal reactivity to this contrast in abused females compared to non-abused females (Fig. 4).

#### Differences in activation based on differences in abuse type and load

There were no significant associations between either hippocampal activity for non-conscious emotions or amygdala activity for conscious emotions and participant abuse type or load (number of abuse type’s experienced).

## Discussion

Our study found experience of abuse during childhood and adolescence to be associated with significantly greater limbic reactivity to both conscious and non-conscious processing of facial emotions later in life. We also observed a double dissociation in limbic reactivity, with differences depending on the timing of early life abuse and whether it impacted conscious or non-conscious emotion processing. Individuals who experienced abuse *in their teens* had heightened amygdala reactivity during conscious processing of all emotions relative to both who were not abused but also compared to those who were abused in their childhood. By contrast, individuals who experienced abuse *prior to 13 years* had greater reactivity in the hippocampus to non-conscious processing, particularly for negative emotions, compared to those without abuse. These findings suggest relative timing of when abuse was experienced early in life seem to manifest differently at the neural level and has implications for conscious vs. non-conscious level of emotion processing.

The hippocampus and amygdala are two distinct limbic brain regions that play important but different roles in emotion processing [40–42]. While the amygdala is responsible for the rapid and automatic detection and processing of emotional stimuli, the hippocampus is more involved in the conscious, contextual, and episodic aspects of emotion processing [41]. These two regions work in concert, with the amygdala providing the initial emotional salience and the hippocampus contributing to the formation and retrieval of emotional memories and the regulation of emotional responses based on contextual information [41]. Disruptions or alterations in the functioning of either region can lead to impairments in emotional processing, emotional memory, and emotional regulation. A higher level of amygdala activation during conscious emotion processing across all emotions could suggest a bias towards heightened emotional reactivity in early life abuse suffers that persists later in life – which accords with this finding being the most robust finding on the impact of childhood abuse on the brain [43]. Notably, in our study, this heightened activation was significant only during the conscious task and specifically in individuals who experienced abuse during adolescence. This suggest that adolescent abuse may primarily affect the indirect amygdala pathway that engages cortical processing. However, it is important to note that we did not observe significant alterations in cortical regions typically associated with emotion regulation in abuse sufferers, such as the medial prefrontal cortex. This could potentially be explained by the nature of our task, which was not designed as an explicit emotion regulation task and may not have fully engaged the cortical regions involved in regulating emotional responses. Additionally, this effect appears to be a common trait mechanism across individuals with depression and stress disorders, rather than being driven by their current symptom state. Interestingly, this heightened amygdala activation was not observed in the anxiety or control groups. While this may reflect true differences, it is also possible that the small sample sizes of adolescent abuse sufferers in these cohorts (9 out of 29 in the anxiety group and 13 out of 212 in the control group) contributed to the lack of significance. Therefore, the absence of this mechanism in these groups cannot be definitively ruled out. Our findings also align with dimensional models of adversity distinguishing threat (e.g., abuse) from deprivation (e.g., neglect, institutionalization). A recent meta-analysis by Hosseini-Kamkar et al. [44] showed that threat-type adversities are reliably associated with amygdala hyperactivation and reduced prefrontal engagement, while deprivation-alone studies showed no consistent effects—likely due to smaller samples and definitional variability. Still, some deprivation studies, particularly involving early institutional care, have reported similar amygdala reactivity, suggesting some overlap in salience-related neural responses [45, 46]. Our findings extend this literature by demonstrating that abuse, as a form of interpersonal threat, shows timing-specific effects on limbic activation: with early abuse linked to heightened hippocampal activation during non-conscious emotion processing, and adolescent abuse associated with amygdala hyperactivation during conscious emotion processing. These results highlight the importance of both adversity type and timing in shaping emotion-processing circuitry.

Neurodevelopmentally, the amygdala is one of the earliest brain regions to develop, with its basic structure and connectivity present at birth [47]. During early childhood, the amygdala exhibits heightened reactivity to emotional stimuli, which is believed to be an adaptive mechanism to facilitate learning about potential threats in the environment [48]. Amygdala reactivity to emotional stimuli remains heightened during childhood and early adolescence, but gradually becomes more regulated and modulated by the developing prefrontal cortex [24, 48]. Previous studies in both animals and humans have demonstrated that abuse suffered during this critical neurodevelopmental period seems to alter the neurodevelopmental pathway of the amygdala, with increased amygdala reactivity to emotional cues persisting later in life and increasing risk for psychopathology [49]. This is true not only for fear or threatening emotional cues [5, 7, 23], but also when processing non-emotional stimuli, suggesting a generalized heightened vigilance or threat sensitivity [16]. We also found an increased amygdala activation in abuse sufferers for not only negative emotional cues but also for positive and neutral facial cues. Our study also demonstrates this heightened amygdala reactivity in individuals who suffered later abuse and not in those who experienced it prior to 13 years, which underscores importance of timing of abuse experienced [1]. This aligns with previous work that has demonstrated differential amygdala engagement with enhanced amygdala response to emotional cues for those who experienced maltreatment during their late teens whereas a blunted amygdala response in those who suffered physical maltreatment during early childhood [50]. While we did not observe a significant blunting effect in early childhood abuse, our null finding for this group is consistent with Sicorello et al. [51], who found no association between amygdala activity and ACEs during the 5–15 year age window. However, the failure to replicate such timing effects in larger samples [52], underscores the complexity and heterogeneity in developmental timing effects and highlights the need for cautious interpretation.

Previous work has also demonstrated altered hippocampal engagement during conscious processing of emotional stimuli due to the experience of early life adversities. For example, interparental violence or peer physical or emotional bullying at 9–10 years has been shown to be associated with a blunted hippocampal response, whereas emotional neglect by the age 14 and parental verbal abuse at ages 15–17 years has been found associated with an enhanced response [21]. An increased activation of the hippocampus during processing of threatening information in youths with a history of caregiver deprivation and emotional neglect during early childhood has also been previously reported [53]. To our knowledge there have been no reports on altered hippocampal activation during non-conscious processing of emotional stimuli due to the experience of childhood abuse. Similar to its role during conscious emotion processing, the hippocampus has been shown to be involved in implicit emotional learning and conditioning, such as context dependent fear conditioning and likely contributes to the formation of non-conscious associations between contextual cues and emotional responses [54, 55]. It also has reciprocal functional connections with the amygdala that may play a role in implicit modulation of emotional responses [56]. Like the amygdala, the hippocampus is one of the first structures that develops in early childhood with basic anatomical structure and connections present at birth [57]. This allows formation of early memories. Throughout childhood and into adolescence, the hippocampus gradually develops more specialized functions related to learning, memory formation, and spatial navigation and by adulthood has fully developed its abilities related to episodic and autobiographical memory formation and retrieval [58]. The hippocampus excels at forming and retrieving these types of complex episodic memories by binding together disparate pieces of information (e.g., sights, sounds, smells, emotions) into cohesive personal experiences [59]. It is possible that the experience of abuse during childhood is encoded to negative emotional cues which are retrieved as an automatic or implicit response to negative stimuli even in later life. This could explain why experiencers of abuse displayed an enhanced hippocampal response to non-conscious but not for conscious processing of negative stimuli relative to those who did not suffer from abuse. This finding was significant in analyses of our whole cohort as well as individuals who experienced abuse preadolescence. As there were no differences in hippocampal activation between individuals who were abused pre vs post adolescence, whether this neural effect is moderated by timing of abuse remains unclear.

Both the amygdala and hippocampal effects were not driven by current depression, anxiety and stress symptoms. While we did not observe a significant diagnosis × abuse interaction for hippocampal activation, such an interaction was present for the amygdala during conscious emotion processing. Post hoc analyses revealed that abuse-related increases in amygdala activation were significant only in the depression and stress groups, but not in those with anxiety disorders or healthy controls. Additionally, we found that individuals with PTSD/stress-related disorders exhibited significantly greater amygdala activation compared to anxiety and control groups across emotional conditions in the adolescent abuse cohort. This pattern suggests that abuse-related amygdala hyperactivity may be a key neural feature of affective and stress-related vulnerability, while its absence in anxity and healthy individuals may reflect more resilient trajectories in neurobiological responses to early adversity. Meanwhile, the hippocampal finding appears more diagnosis-independent, suggesting a potential neural signature of early abuse exposure that is not specific to clinical status.

The following limitations should be considered in the context of the study. Firstly, our analysis relies on retrospective measures of childhood adversities which may not have been sufficiently sensitive to identify detailed histories of the nature, timing, and extent of abuse. Unfortunately, this is a common limitation of cross-sectional retrospective studies and can only be tackled with detailed long-term longitudinal cohorts. Our small subgroups based on abuse type and amount of trauma may have limited ability to detect these effects in our analysis. It is possible that more detailed measurement of abuse and timing, such as the Maltreatment and Abuse Chronology of Exposure Scale [60], may allow interrogating more nuanced aspects of the timing and nature of adverse childhood experiences. We also did not assess childhood neglect, which has also been found associated with distinct patterns of brain changes. The absence of data on adult trauma or abuse limits our ability to distinguish the neural effects of childhood abuse from those potentially associated with later-life adversity. Our cohort was limited to individuals with depression, anxiety and stress disorders. Including patients with bipolar, schizophrenia, borderline personality and attention disorders would have helped to generalize findings across several psychiatric disorders. Nevertheless, our findings point to possibly transdiagnostic effects with depression, anxiety and stress disorders each of which have been associated with experience of childhood adversities. While our analytic approach was guided by established models distinguishing between conscious and non-conscious emotion processing - typically associated with indirect (cortical) and direct (subcortical) amygdala pathways respectively, we acknowledge that recent theoretical work has challenged this dual-route framework. In particular, a more integrated, network-based model in which emotion processing emerges from flexible, distributed interactions rather than anatomically discrete circuits has been proposed [61]. Our use of predefined regions of interest was well suited to test classical hypotheses of functional activation differences, but future studies using whole-brain and functional connectivity analyses may be better positioned to evaluate these emerging models of distributed emotion processing.

In summary, our findings provide new insights into how the timing and nature of childhood and adolescent abuse shape long-term neural processing of emotions across psychiatric disorders. The double dissociation we observed in limbic reactivity where amygdala activation was heightened during conscious emotion processing in adolescent abuse sufferers and hippocampal activation was elevated during non-conscious processing in those abused during childhood—underscores the importance of considering both the timing of abuse and the level of emotional processing in understanding its impact on brain function. These results point to distinct neural mechanisms that may underlie the heightened emotional sensitivity and reactivity observed in abuse survivors, with potential implications for tailored interventions targeting different stages of development.

## Supplementary information

Supplementary Material

## Data Availability

Data is not publicly available due to ethical restrictions; however, we are happy to make the data available upon request.
