# Supplementary material for: Emotional scars: limbic brain processing alterations in adults with childhood abuse across mental health disorders
Source: Mol Psychiatry. 2026 Mar 5;31(7):3945–54. doi: 10.1038/s41380-026-03511-9 (PMC13269135; doi:10.1038/s41380-026-03511-9)
Supplement: Supplementary file 1 — Supplementary Material [file 41380_2026_3511_MOESM1_ESM.docx]

Supplementary Materials

**Working title: Alterations to emotional processing in adults with a history of child abuse**

Mayuresh S. Korgaonkar^1,2^, Cheryl Tobler^1,3^, Kim Felmingham PhD^4^, Leanne M. Williams, PhD^5,6^, Richard A. Bryant, PhD^1,3^, Isabella A. Breukelaar^1,3^

1. Brain Dynamics Centre, Westmead Institute for Medical Research, University of Sydney, Westmead, NSW, Australia
2. Discipline of Psychiatry, Sydney Medical School, Westmead, NSW, Australia
3. School of Psychology, University of New South Wales, Sydney, Australia
4. Discipline of Psychological Science, University of Melbourne
5. Department of Psychiatry and Behavioral Sciences, Stanford University
6. Sierra-Pacific Mental Illness Research, Education, and Clinical Center (MIRECC) VA Palo Alto Health Care System, Palo Alto

# **S1: Supplementary Methods**

**Table S1.** Summary of contributing studies

| Study Name | Number of Participants in Analysis | Study Criteria | Data collected | References |
| --- | --- | --- | --- | --- |
| International Study to Predict Optimized Treatment for Depression (iSPOT-D) | 257 | Inclusion:   - Age 18-65 - Fluent and literate in English - Provide written informed consent. - HRSD17 ≤ 16 - Meets DSM-IV criteria for single or recurrent nonpsychotic MDD (using MINI plus)   Exclusion:   - Score of ≥ 8 on Section C of MINI Plus (actively suicidal) - History of bipolar disorder, schizophrenia, schizoaffective disorder, or psychosis - Current primary diagnosis of anorexia/bulimia, OCD, PTSD - Known contraindication to escitalopram, sertraline, venlafaxine or previous treatment failure at highest dose - Taking other contraindicated medications - Use of non-protocol antidepressant or CNS that cannot be washed out - General medical condition contraindicative to antidepressant treatment or protocol - Substance dependence - Brain injury or blow to head that result in loss of consciousness for greater than five minutes. - Severe impediment to vision, hearing or hand movement that is likely to interfere to comprehension or completion of assessment. - Pregnant or breast-feeding - Participant in investigational study within four months that could impact MDD symptoms. | Interview:   - **Mini International Neuropsychiatric Interview, a structured interview with DSM-IV criteria.** - 17-item & 21-item Hamilton Rating Scale for Depression (HRSD17)   Web Self-report (WebQ):   - **Demographic Medical History** - **Depression, Anxiety and Stress Scale (DASS).** - **Early Life Stress Questionnaire (ELSQ)** - 16-item Quick Inventory of Depressive Symptomatology (QIDS_16_)   MRI (3.0T GE SignaTwinspeed, Westmead):   - **T1 SPGR** - DTI - **fMRI tasks:** Oddball, CPT, Go-NoGo, unmasked conscious emotion processing, masked non-conscious emotion processing | Williams, L.M., Rush, A.J., Koslow, S.H. *et al.* International Study to Predict Optimized Treatment for Depression (iSPOT-D), a randomized clinical trial: rationale and protocol. *Trials* **12**, 4 (2011). <https://doi.org/10.1186/1745-6215-12-4>  Grieve, S.M., Korgaonkar, M.S., Etkin, A. *et al.* Brain imaging predictors and the international study to predict optimized treatment for depression: study protocol for a randomized controlled trial. *Trials* **14**, 224 (2013). <https://doi.org/10.1186/1745-6215-14-224>  ClinicalTrials.gov ID: NCT00693849; |
| NHMRC Centre for Clinical Research Excellence in Anxiety Disorders (CCRE) | 250 | Inclusion:   - Age 18-65 - Fluent and literate in English - Provide written informed consent. - Meets DSM-IV criteria for mood or anxiety disorder (using MINI 5.0) or healthy control. - Medication permitted as long as dosage had been stable for previous two months.   Exclusion:   - History of bipolar disorder, schizophrenia, schizoaffective disorder, or psychosis - General medical condition contraindicative to antidepressant treatment or protocol - Substance dependence - Brain injury or blow to head that result in loss of consciousness for greater than five minutes. - Severe impediment to vision, hearing or hand movement that is likely to interfere to comprehension or completion of assessment. - Pregnant | Interview:   - **Mini International Neuropsychiatric Interview, a structured interview with DSM-IV criteria.** - Beck Depression Investory-2 (BDI) - Clinician Administered Posttraumatic Stress Disorder Scale (CAPS)   Web Self-report (WebQ):   - **Demographic Medical History** - **Depression, Anxiety and Stress Scale (DASS).** - **Early Life Stress Questionnaire (ELSQ)**   MRI (3.0T GE SignaTwinspeed, Westmead):   - **T1 SPGR** - DTI - **fMRI tasks:** Emotional Reappriasal Task x2, Go-NoGo, unmasked conscious emotion processing, masked non-conscious emotion processing. | R. A. Bryant *et al.*, Reappraisal-related neural predictors of treatment response to cognitive behavior therapy for post-traumatic stress disorder. *Psychol Med* **51**, 2454-2464 (2021).  <https://doi.org/10.1017/S0033291720001129> |
| **Limbic maturational changes in adolescence and young adulthood (LIMCA): a Longitudinal Study** | 128 | Inclusion:   - Age 8-38 years* - Fluent and literate in English - Provide written informed consent. - Willing and available to be retested 1.5 years later   Exclusion:   - Current or previous diagnosis of mental illness - General medical condition (i.e. thyroid, heart disease, cancer, genetic disorder, blood borne disease). - Brain injury or blow to head that result in loss of consciousness for greater than five minutes. - Severe impediment to vision, hearing or hand movement that is likely to interfere to comprehension or completion of assessment. - Pregnant or breastfeeding   *only participants over 18 included in the current analysis | Interview:   - Phone screening for inclusion and exclusion criteria   Web Self-report (WebQ):   - **Demographic Medical History** - **Depression, Anxiety and Stress Scale (DASS).** - **Early Life Stress Questionnaire (ELSQ)**   MRI (3.0T GE SignaTwinspeed, Westmead):   - **T1 SPGR** - DTI - **fMRI tasks:** Oddball, CPT, Go-NoGo, unmasked conscious emotion processing, masked non-conscious emotion processing | Breukelaar, I .A. *et al.* Cognitive ability is associated with changes in the functional organization of the cognitive control brain network. *Human Brain Mapping* **39**, 5028–5038 (2018). DOI: 10.1002/hbm.24342  Breukelaar, I. A. *et al.* Cognitive control network anatomy correlates with neurocognitive behavior: A longitudinal study. *Human Brain Mapping* **38**, 631–643 (2017). DOI: 10.1002/hbm.23401 |

*Common measures used in this analysis shown in* ***bold***

## **S.1.1 fMRI acquisition**

Functional MRI data was acquired using an echo planar imaging sequence with the following parameters: repetition time (TR)=2,500ms, echo time (TE)=27.5ms, matrix=64x64, field of view (FOV)=24cm, flip angle=90°, 120 volumes with a total scan time of 5min and 8s. Forty slices, each 3.5mm thick, covered the whole brain in each volume. Three dummy scans were also acquired at the start of every acquisition to allow magnetization to stabilize to steady state. T1-weighted images were acquired in the sagittal plane using a 3D SPGR sequence (FOV=256mm, TR=8.3ms; TE=3.2ms; flip angle=11°; TI=500ms; NEX=1 and ASSET=1.5; frequency direction: S/I; 256x256 matrix, 180 contiguous slices, 1mm isotropic voxels) and used for normalization of the functional data.

**S.1.2 fMRI preprocessing and first level analysis**

The fMRI data was preprocessed and analyzed using SPM8 software (www.fil.ion.ucl.ac.uk/spm). Motion correction was performed by realigning and unwarping the fMRI images to the first image of each task run. Following realignment and unwarping, quality control diagnostics were completed on the time series data for each run. Data volumes that were associated with extreme 1) movement (framewise displacement from one time point to the next) and 2) changes in BOLD signal intensity (as indexed by the mean squared difference in signal intensity over the entire volume from one time point to the next divided by the mean signal across the volume averaged across the full time series) were censored (temporally masked) to reduce the influence of motion and related artifacts. Framewise displacement was calculated as the sum of the absolute values of the differentiated realignment estimates as in Power et al., 2014. Volumes were censored using established thresholds of framewise displacement greater than or equal to 0.3mm and scaled signal intensity differences greater than 10)(TSDiffana refs; Achaibou, 2015; Power, 2012; Power, 2014; Siegal, 2014). Censoring was implemented with the time series difference analysis toolbox http://www.fil.ion.ucl.ac.uk/spm/ext/# TSDiffAna[1]  and in house scripts. A temporal mask was then created for each censored volume (as well as subsequent volume) and used as regressors of no interest in the first level statistical models (Power, 2012; Power 2014). For normalization to stereotactic MNI space, the T1- weighted data were normalized to standard space using the FMRIB nonlinear registration tool and the fMRI EPI data were coregistered to the T1 data using FMRIB linear registration tool (Andersson et al, 2007a,b). Normalization warps from these two steps were stored for use in functional to standard space transformations. Global signal was estimated using a eroded mask within the ventricles and white matter and was removed from the motion-corrected fMRI time series. fMRI data was smoothed using an 8 mm Gaussian kernel and high-pass filtered using a cutoff period of 128s.

## **Table s2. Task Parameters**

| *Emotion tasks* | | | | | |
| --- | --- | --- | --- | --- | --- |
| Unmasked Conscious Emotion Processing Task ([Bryant et al, 2008](https://www-nature-com.ezproxy.library.sydney.edu.au/articles/npp2012252#ref-CR7); [Williams et al, 2006a](https://www-nature-com.ezproxy.library.sydney.edu.au/articles/npp2012252#ref-CR44), [2006b](https://www-nature-com.ezproxy.library.sydney.edu.au/articles/npp2012252#ref-CR45)) | Explicit processing of emotions at the level of conscious awareness | A standardized set of 3D evoked facial expressions (fear, anger, disgust, sadness, happiness, neutral) ([Gur et al, 2002](https://www-nature-com.ezproxy.library.sydney.edu.au/articles/npp2012252#ref-CR19)), modified to be centrally positioned at eye level. | Each face presented for 500 ms, with an interstimulus interval of 750 ms | Pay attention to each emotion face in order to respond to post-testing questions. | A total of 240 stimuli. Stimuli were grouped in blocks of 8 faces of the same emotion, with each emotion block repeated 5 times and presented in pseudorandom order. One volume per 2 stimuli was acquired. |
| Masked Non-Conscious Emotion Processing Task ([Bryant et al, 2008](https://www-nature-com.ezproxy.library.sydney.edu.au/articles/npp2012252#ref-CR7); [Williams et al, 2006a](https://www-nature-com.ezproxy.library.sydney.edu.au/articles/npp2012252#ref-CR44), [2006b](https://www-nature-com.ezproxy.library.sydney.edu.au/articles/npp2012252#ref-CR45)) | Implicit processing of emotions below the level of conscious awareness | The same set of facial emotion stimuli as above, presented in a backward-masking design to prevent awareness. | Each face presented briefly (10 ms), followed immediately by a neutral face mask stimulus for 150 ms. | Pay attention to each emotion face. | A total of 240 stimuli. Stimuli were grouped in blocks of eight faces of the same emotion, with each emotion block repeated 5 times and presented in pseudorandom order. One volume per 2 stimuli was acquired. |

**
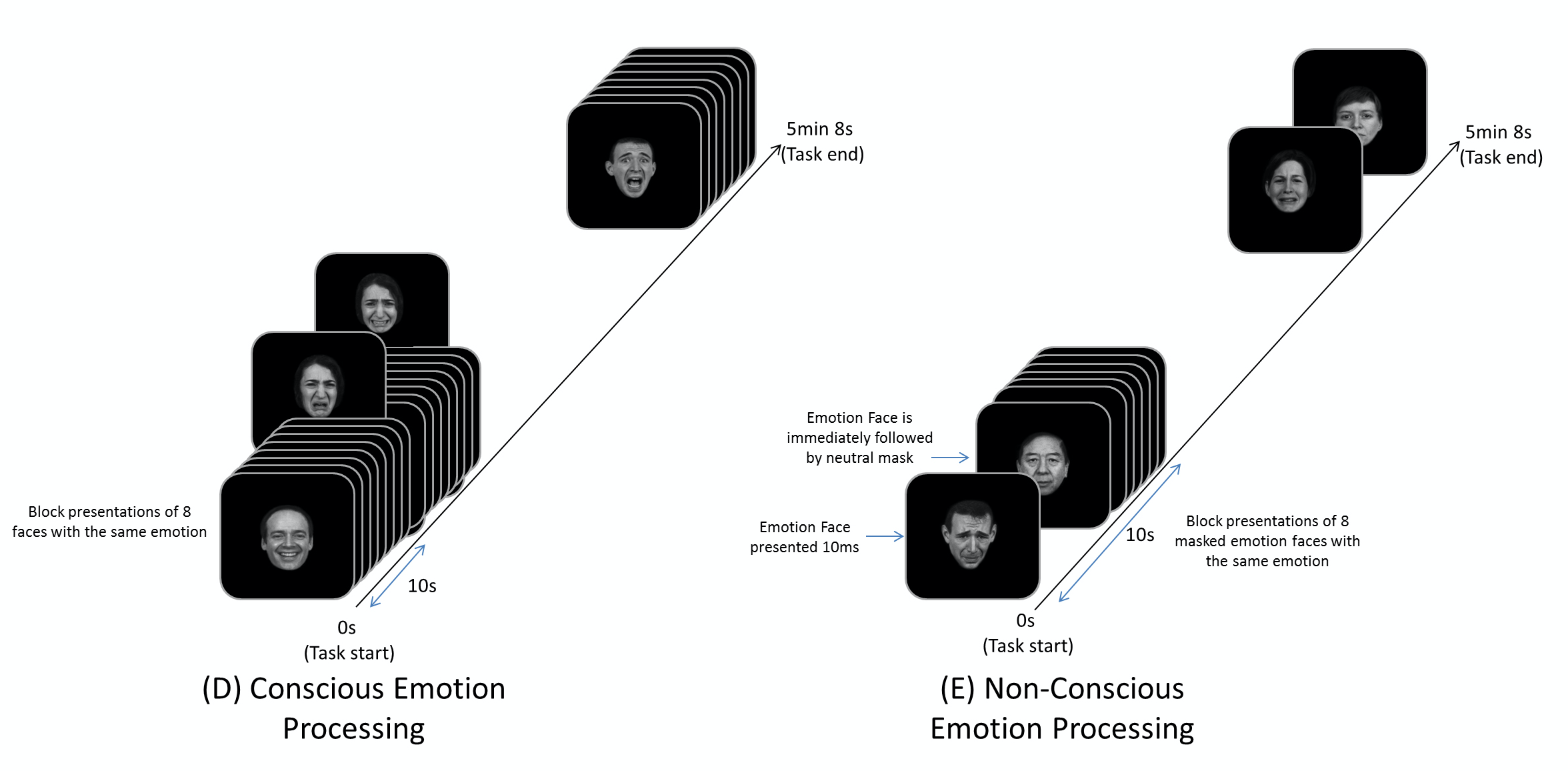
**

**Figure s1.** Conscious Emotion Processing and Non-conscious Emotion Processing tasks.

# **S2: Supplementary Results**

**Table s3. Percentage of overlap between clinical diagnostic categories**

| **Diagnostic Cat** | **MDD%** | **Anxiety%** | **PTSD%** |
| --- | --- | --- | --- |
| **MDD** | **NA** | **54.5** | **0** |
| **Anxiety** | **55.4** | **NA** | **0** |
| **PTSD** | **71** | **83.9** | **NA** |

**Figure s2. Venn diagram illustrating overlap across diagnostic categories**

**Table s4. Results of non-conscious ANCOVA group comparison, for all possible contrasts between facial emotions controlling for age, sex, years of education, and diagnosis as well as additional ANCOVA results also controlling for mean fractional displacement (FD) using extracted mean activation from significant hippocampal cluster.**

|  | **No Abuse** | **Abuse** | **Primary ANCOVA** | | **Controlling mean FD** | |  |
| --- | --- | --- | --- | --- | --- | --- | --- |
|  | Mean (beta) Activation | Mean (beta) Activation | **F value** | **P value** | **F value** | **P value** |  |
| Anger v Disgust | 0.009 | -0.004 | 0.83 | 0.362 | 0.897 | 0.344 |  |
| Anger v Fear | 0.002 | 0.007 | 1.16 | 0.283 | 1.250 | 0.264 |  |
| Anger v Happy | -0.009 | 0.013 | 7.06 | 0.008 | 7.050 | 0.008 | * |
| Anger v Sad | -0.002 | -0.007 | 0.01 | 0.905 | 0.018 | 0.893 |  |
| Anger v Neutral | -0.001 | 0.025 | 9.36 | 0.002 | 9.690 | 0.002 | * |
| Disgust v Fear | -0.006 | 0.011 | 3.91 | 0.049 | 4.230 | 0.040 |  |
| Disgust v Happy | -0.017 | 0.017 | 11.00 | 0.001 | 11.200 | 0.001 | ** |
| Disgust v Sad | -0.011 | -0.003 | 0.58 | 0.447 | 0.606 | 0.437 |  |
| Disgust v Neutral | -0.009 | 0.029 | 19.30 | 0.000 | 20.300 | 0.000 | *** |
| Fear v Happy | -0.011 | 0.006 | 1.98 | 0.160 | 1.860 | 0.173 |  |
| Fear v Sad | -0.005 | -0.014 | 1.51 | 0.219 | 1.660 | 0.197 |  |
| Fear v Neutral | -0.003 | 0.018 | 3.68 | 0.055 | 3.700 | 0.055 |  |
| Happy v Sad | 0.007 | -0.020 | 6.30 | 0.012 | 6.370 | 0.012 | * |
| Happy v Neutral | 0.008 | 0.012 | 0.23 | 0.634 | 0.275 | 0.600 |  |
| Sad v Neutral | 0.001 | 0.032 | 9.23 | 0.002 | 9.660 | 0.002 | ** |

**Table s5. Test of results with and without clinical group included in the model, as an interaction term and the association of clinical group with hippocampal activation for non-conscious processing not including abuse grouping.**

|  | **Model 1 – primary model** | | **Model 2 - no clinical group** | | **Model 3 - interaction** | | **Model 4 - only clinical group** | | |
| --- | --- | --- | --- | --- | --- | --- | --- | --- | --- |
|  | **F value** | **P value** | **F value** | **P value** | **F value** | **P value** | **F value** | **P value** |  |
| Anger-Happy | 7.06 | 0.008* | 5.74 | 0.017* | 1.06 | 0.395 | 0.92 | 0.514 |  |
| Anger-Neutral | 9.36 | 0.002* | 7.67 | 0.006* | 1.15 | 0.319 | 0.961 | 0.477 |  |
| Disgust-Happy | 11 | 0.001** | 11.9 | 0.001** | 1.36 | 0.194 | 1.44 | 0.159 |  |
| Disgust- Neutral | 19.3 | 0.000** | 20.5 | 0.000** | 0.792 | 0.637 | 0.875 | 0.557 |  |
| Fear-Happy | 1.98 | 0.16 | 2.83 | 0.093 | 1.3 | 0.229 | 1.37 | 0.188 |  |
| Fear-Neutral | 3.68 | 0.055^ | 4.7 | 0.031***** | 0.819 | 0.611 | 0.913 | 0.52 |  |
| Happy-Sad | 6.3 | 0.012* | 5.39 | 0.021* | 1.23 | 0.268 | 1.13 | 0.339 |  |
| Sad-Neutral | 9.23 | 0.002* | 7.85 | 0.005* | 1.74 | 0.069 | 1.55 | 0.117 |  |

Model 1: Var ~ Sex + Age + YrsEdu + ClinicalGroup + Abuse

Model 2: Var ~ Sex + Age + YrsEdu + Abuse

Model 3: Var ~ Sex + Age + YrsEdu + ClinicalGroup * Abuse

Model 4: Var ~ Sex + Age + YrsEdu + ClinicalGroup

“Var” represents extracted mean hippocampal activation for that contrast

**Table s6. Comparison of hippocampal activation at relevant contrasts between abuse groups with differing onset ages**

|  | Abuse under 18 vs no Abuse | | Abuse under 13 vs No abuse | | Abuse over 13 vs No abuse | | Abuse over vs under 13 | |
| --- | --- | --- | --- | --- | --- | --- | --- | --- |
| Variable | FStat | P.value | FStat | P.value | FStat | P.value | FStat | P.value |
| AngervHappy | 7.06 | 0.008* | 5.41 | 0.02* | 2.74 | 0.0988 | 0.0229 | 0.88 |
| AngervNeutral | 9.36 | 0.002* | 11.5 | <0.001** | 1.58 | 0.209 | 1.95 | 0.163 |
| DisgustvHappy | 11 | <0.001** | 7.19 | 0.008* | 5.04 | 0.025* | 0.01 | 0.921 |
| DisgustvNeutral | 19.3 | <0.001** | 18.8 | <0.001** | 5.31 | 0.022* | 2.97 | 0.086 |
| FearvHappy | 1.98 | 0.16 | 0.185 | 0.668 | 2.81 | 0.094 | 0.793 | 0.374 |
| FearvNeutral | 3.68 | 0.0554 | 2.73 | 0.0992 | 1.91 | 0.168 | 0.302 | 0.583 |
| SadvHappy | 6.3 | 0.012* | 2.99 | 0.0845 | 4.66 | 0.031* | 0.682 | 0.41 |
| SadvNeutral | 9.23 | 0.002* | 8.53 | 0.004* | 3.75 | 0.0533 | 0.326 | 0.568 |

**Table s7. Results of ANCOVA group comparison between participants abused after age 13, before age 13 and not at all, for each of the emotions, using extracted significant amygdala cluster from SPM analysis**

|  | ANCOVA; | | TukeyHSD Post-Hoc | | | | | |
| --- | --- | --- | --- | --- | --- | --- | --- | --- |
|  | Sex, Diagnosis, Age, YrsEdu | | 13to17-None | | 13to17-0to12 | | 0to12-None | |
|  | F value | P value | MeanDiff | P-adj | MeanDiff | P-adj | MeanDiff | P-adj |
| Anger | 8.428 | 0.000 | 0.205 | 0.000 | 0.227 | 0.000 | 0.022 | 0.839 |
| Disgust | 9.873 | 0.000 | 0.233 | 0.000 | 0.256 | 0.000 | 0.058 | 0.828 |
| Fear | 8.466 | 0.000 | 0.195 | 0.002 | 0.253 | 0.000 | 0.154 | 0.332 |
| Happy | 7.808 | 0.000 | 0.202 | 0.000 | 0.212 | 0.001 | 0.010 | 0.962 |
| Neutral | 9.596 | 0.000 | 0.228 | 0.000 | 0.250 | 0.000 | 0.022 | 0.847 |
| Sad | 8.107 | 0.000 | 0.213 | 0.000 | 0.220 | 0.001 | 0.007 | 0.984 |

**Table s8. Test of results with and without clinical group included in the model, as an interaction term and the association of clinical group with amygdala activation for conscious emotion processing not including abuse grouping.**

|  | **No Abuse** | | | **Over 13 Abuse** | | **Model 1** | | | **Model 2 - no clinical group** | | | | **Model 3 - interaction** | | | | |  |
| --- | --- | --- | --- | --- | --- | --- | --- | --- | --- | --- | --- | --- | --- | --- | --- | --- | --- | --- |
|  | | | **Mean (beta) Activation** | **Mean (beta) Activation** | | **F value** | | **P value** | | | **F value** | **P value** | | **F value** | | **P value** |  |  |
| Anger | | | 0.075 | 0.280 | | 19.100 | | 0.000 | | | 15.700 | 0.000 | | 19.500 | | 0.000 |  |  |
| Disgust | | | 0.064 | 0.296 | | 22.100 | | 0.000 | | | 19.000 | 0.000 | | 22.700 | | 0.000 |  |  |
| Fear | | | 0.069 | 0.264 | | 15.600 | | 0.000 | | | 12.600 | 0.000 | | 16.000 | | 0.000 |  |  |
| Happy | | | 0.053 | 0.254 | | 19.200 | | 0.000 | | | 15.200 | 0.000 | | 19.500 | | 0.000 |  |  |
| Neutral | | | 0.031 | 0.259 | | 19.300 | | 0.000 | | | 16.900 | 0.000 | | 19.700 | | 0.000 |  |  |
| Sad | | | 0.060 | 0.273 | | 20.700 | | 0.000 | | | 16.000 | 0.000 | | 21.100 | | 0.000 |  |  |
|  | | **Abuse before 13** | | | **Over 13 Abuse** | | **Model 1** | | | **Model 2 - no clinical group** | | | | | **Model 3 - interaction** | | | |
|  | | | **Mean (beta) Activation** | **Mean (beta) Activation** | | **F value** | | **P value** | | | **F value** | **P value** | | **F value** | | **P value** |  |  |
| Anger | | | 0.053 | 0.280 | | 12.600 | | 0.000 | | | 14.400 | 0.000 | | 12.900 | | 0.000 |  |  |
| Disgust | | | 0.040 | 0.296 | | 11.000 | | 0.001 | | | 14.400 | 0.000 | | 11.200 | | 0.001 |  |  |
| Fear | | | 0.011 | 0.264 | | 10.400 | | 0.001 | | | 13.800 | 0.000 | | 10.800 | | 0.001 |  |  |
| Happy | | | 0.043 | 0.254 | | 8.680 | | 0.004 | | | 11.200 | 0.001 | | 8.860 | | 0.003 |  |  |
| Neutral | | | 0.009 | 0.259 | | 11.500 | | 0.001 | | | 14.400 | 0.000 | | 11.700 | | 0.001 |  |  |
| Sad | | | 0.054 | 0.273 | | 9.640 | | 0.002 | | | 12.100 | 0.001 | | 9.800 | | 0.002 |  |  |

Model 1: Var ~ Sex + Age + YrsEdu + ClinicalGroup + Abuse Onset

Model 2: Var ~ Sex + Age + YrsEdu + Abuse Onset

Model 3: Var ~ Sex + Age + YrsEdu + ClinicalGroup * Abuse Onset

“Var” represents extracted mean amygdala activation for that contrast

**Table s9. Interaction effects - pair-wise post-hoc comparisons of diagnostic category by prepubertal abuse onset for amygdala responsiveness to each emotion**

|  |  | *pairwise post-hoc comparison p-value* | | | | | |
| --- | --- | --- | --- | --- | --- | --- | --- |
| Comparison | Abuse Onset | ANGER | DISGUST | FEAR | HAPPY | NEUTRAL | SAD |
| Anxiety-Control | 0to12 | 0.905 | 0.958 | 1.000 | 1.000 | 0.990 | 0.972 |
| Anxiety-MDD | 0to12 | 0.530 | 0.274 | 0.416 | 0.735 | 0.857 | 0.369 |
| Anxiety-PTSD | 0to12 | 0.705 | 0.799 | 0.938 | 0.987 | 0.992 | 0.815 |
| Control-MDD | 0to12 | 0.857 | 0.372 | 0.263 | 0.520 | 0.466 | 0.456 |
| Control-PTSD | 0to12 | 0.964 | 0.961 | 0.939 | 0.968 | 0.879 | 0.951 |
| MDD-PTSD | 0to12 | 0.993 | 0.690 | 0.615 | 0.814 | 0.917 | 0.803 |
| Anxiety-Control | 13to17 | 1.000 | 0.952 | 0.781 | 0.947 | 0.960 | 0.966 |
| Anxiety-MDD | 13to17 | 0.347 | 0.405 | 0.812 | 0.653 | 0.483 | 0.746 |
| Anxiety-PTSD | 13to17 | 0.010* | 0.040* | 0.021* | 0.025* | 0.034* | 0.042* |
| Control-MDD | 13to17 | 0.207 | 0.047* | 0.082 | 0.139 | 0.079 | 0.250 |
| Control-PTSD | 13to17 | 0.007* | 0.013* | 0.003* | 0.007* | 0.012* | 0.016* |
| MDD-PTSD | 13to17 | 0.053 | 0.155 | 0.038* | 0.066 | 0.119 | 0.088 |
| Anxiety-Control | None | 0.126 | 0.313 | 0.207 | 0.069 | 0.126 | 0.016* |
| Anxiety-MDD | None | 0.823 | 0.991 | 0.936 | 0.743 | 0.692 | 0.446 |
| Anxiety-PTSD | None | 0.226 | 0.399 | 0.349 | 0.090 | 0.129 | 0.059 |
| Control-MDD | None | 0.112 | 0.068 | 0.091 | 0.074 | 0.264 | 0.063 |
| Control-PTSD | None | 0.943 | 0.953 | 0.970 | 0.811 | 0.805 | 0.909 |
| MDD-PTSD | None | 0.405 | 0.373 | 0.449 | 0.209 | 0.330 | 0.292 |

**Table s10. Interaction effects - pair-wise post-hoc comparisons of prepubertal abuse by diagnostic category for amygdala responsiveness to each emotion**

|  |  | *pairwise post-hoc comparison p-value* | | | | | |
| --- | --- | --- | --- | --- | --- | --- | --- |
| Comparison | Diagnostic Category | ANGER | DISGUST | FEAR | HAPPY | NEUTRAL | SAD |
| 0to12-13to17 | Anxiety | 0.677 | 0.464 | 0.357 | 0.697 | 0.770 | 0.435 |
| 0to12-None | Anxiety | 0.993 | 0.969 | 0.999 | 0.662 | 0.682 | 0.735 |
| 13to17-None | Anxiety | 0.582 | 0.529 | 0.323 | 0.269 | 0.344 | 0.138 |
| 0to12-13to17 | Control | 0.877 | 0.936 | 0.920 | 0.941 | 0.871 | 0.844 |
| 0to12-None | Control | 0.483 | 0.289 | 0.098 | 0.384 | 0.274 | 0.370 |
| 13to17-None | Control | 0.982 | 0.835 | 0.651 | 0.886 | 0.909 | 0.971 |
| 0to12-13to17 | MDD | 0.006* | 0.009* | 0.037* | 0.033* | 0.006* | 0.058 |
| 0to12-None | MDD | 0.343 | 0.065 | 0.174 | 0.090 | 0.281 | 0.063 |
| 13to17-None | MDD | 0.000** | 0.000** | 0.000** | 0.000** | 0.000** | 0.000** |
| 0to12-13to17 | PTSD | 0.002* | 0.004* | 0.001* | 0.002* | 0.004* | 0.004* |
| 0to12-None | PTSD | 0.690 | 0.579 | 0.448 | 0.441 | 0.492 | 0.586 |
| 13to17-None | PTSD | 0.007* | 0.019* | 0.006* | 0.014* | 0.020* | 0.018* |


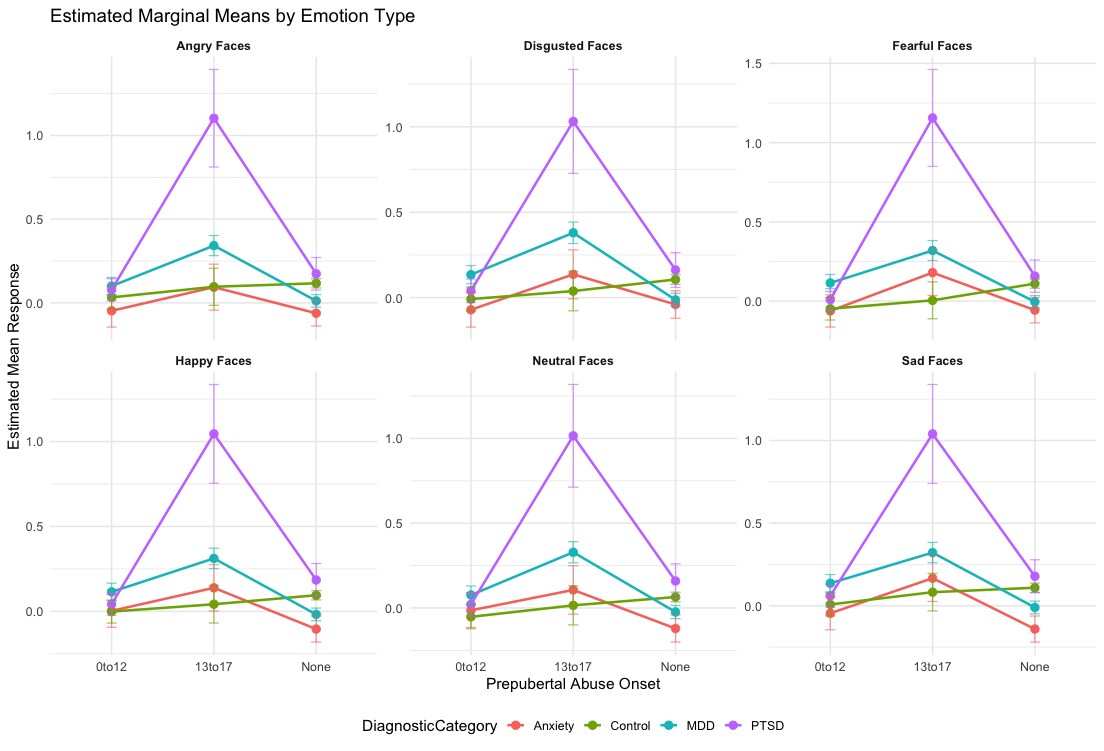
 **Figure s3. Interaction plots of each emotion across each prepubertal abuse onset for each diagnostic category using estimated mean response of amygdala to conscious emotion processing.**
